# Supplementary material for: Evaluation of Platelet-Derived Extracellular Vesicles in Gingival Fibroblasts and Keratinocytes for Periodontal Applications
Source: Int J Mol Sci. 2022 Jul 11;23(14):7668. doi: 10.3390/ijms23147668 (PMC9321144; doi:10.3390/ijms23147668)
Supplement: Supplementary file 1 [file ijms-23-07668-s001.zip › ijms-1799213-supplementary.pdf]

Supplementary information

a

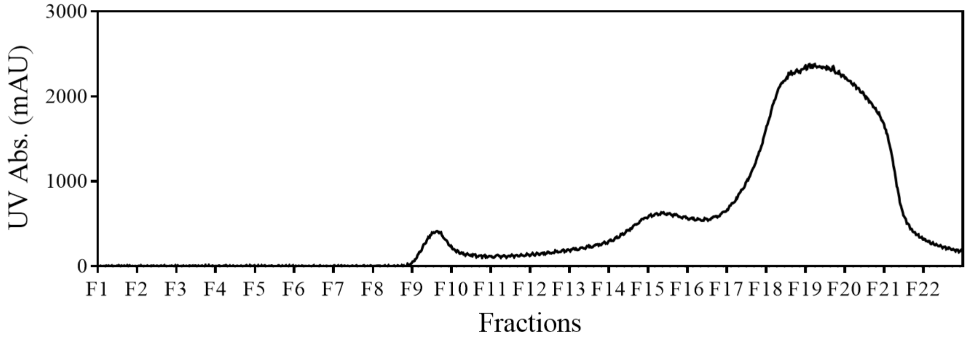

b

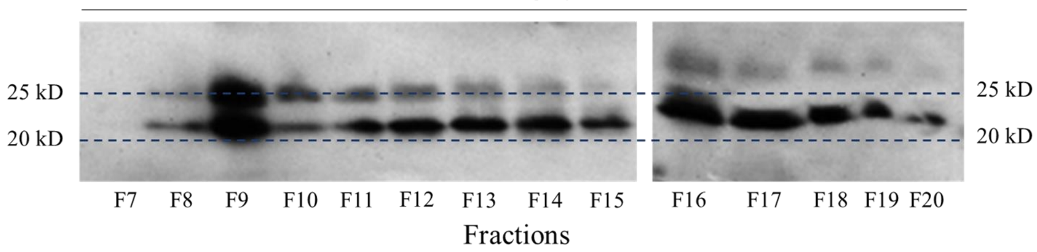

Supplementary Figure S1. Size Exclusion Chromatography (SEC) fractions. **(a)** Ultraviolet absorbance per fraction, correlated to protein presence. **(b)** Presence of CD9 per fraction, the same amount of volume was loaded per well.

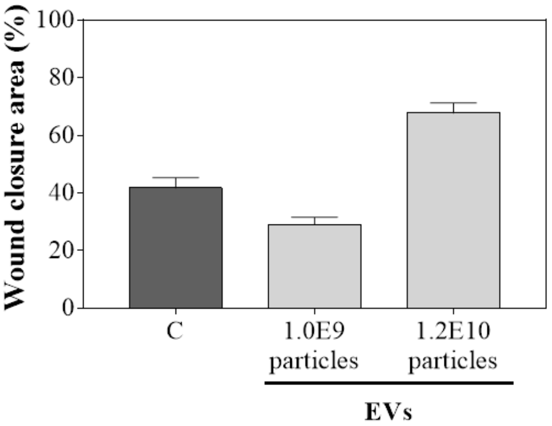

Supplementary Figure S2. Dose-response evaluation. EVs dose response evaluated on ihGF by a wound healing assay. Two different doses, 1.0E9 and 1.2E10 particles, were tested. 1.2E 10 particles are equivalent to 5 ug of EVs.

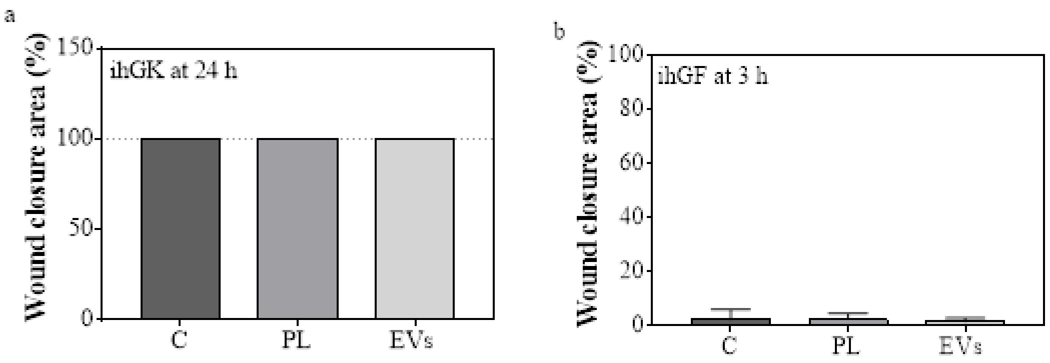

Supplementary Figure S3. Wound closure area. **(a)** Wound closure area after 24 h of treatment in ihGK. **(b)** Wound closure area after 3 h of treatment in ihGF.
